# Inhibition of Nuclear Factor of Activated T-Cells (NFAT) Suppresses Accelerated Atherosclerosis in Diabetic Mice

**DOI:** 10.1371/journal.pone.0065020

**Published:** 2013-06-03

**Authors:** Anna V. Zetterqvist, Lisa M. Berglund, Fabiana Blanco, Eliana Garcia-Vaz, Maria Wigren, Pontus Dunér, Anna-Maria Dutius Andersson, Fong To, Peter Spegel, Jan Nilsson, Eva Bengtsson, Maria F. Gomez

**Affiliations:** Department of Clinical Sciences in Malmö, Lund University, Malmö, Sweden; Baker IDI Heart and Diabetes Institute, Australia

## Abstract

**Objective of the Study:**

Diabetic patients have a much more widespread and aggressive form of atherosclerosis and therefore, higher risk for myocardial infarction, peripheral vascular disease and stroke, but the molecular mechanisms leading to accelerated damage are still unclear. Recently, we showed that hyperglycemia activates the transcription factor NFAT in the arterial wall, inducing the expression of the pro-atherosclerotic protein osteopontin. Here we investigate whether NFAT activation may be a link between diabetes and atherogenesis.

**Methodology and Principal Findings:**

Streptozotocin (STZ)-induced diabetes in apolipoprotein E^−/−^ mice resulted in 2.2 fold increased aortic atherosclerosis and enhanced pro-inflammatory burden, as evidenced by elevated blood monocytes, endothelial activation- and inflammatory markers in aorta, and pro-inflammatory cytokines in plasma. *In vivo* treatment with the NFAT blocker A-285222 for 4 weeks completely inhibited the diabetes-induced aggravation of atherosclerosis, having no effect in non-diabetic mice. STZ-treated mice exhibited hyperglycemia and higher plasma cholesterol and triglycerides, but these were unaffected by A-285222. NFAT-dependent transcriptional activity was examined in aorta, spleen, thymus, brain, heart, liver and kidney, but only augmented in the aorta of diabetic mice. A-285222 completely blocked this diabetes-driven NFAT activation, but had no impact on the other organs or on splenocyte proliferation or cytokine secretion, ruling out systemic immunosuppression as the mechanism behind reduced atherosclerosis. Instead, NFAT inhibition effectively reduced IL-6, osteopontin, monocyte chemotactic protein 1, intercellular adhesion molecule 1, CD68 and tissue factor expression in the arterial wall and lowered plasma IL-6 in diabetic mice.

**Conclusions:**

Targeting NFAT signaling may be a novel and attractive approach for the treatment of diabetic macrovascular complications.

## Introduction

A much more widespread and aggressive form of atherosclerosis is observed in the coronary arteries, lower extremities and extracranial carotid arteries of diabetic patients, causing nearly 80% of all deaths and much of their disability [1]. Both diabetes type 1 and type 2 are independent risk factors for myocardial infarction, peripheral vascular disease and stroke. Despite vast clinical experience linking diabetes and atherosclerosis, it is still unclear how diabetes accelerates the clinical course of the disease. A wealth of epidemiologic evidence demonstrate that hyperglycemia increases cardiovascular event rates and worsens outcome [2]. Recent studies also show a causal association between elevated glucose levels and increased carotid intima-media thickness, a surrogate marker of subclinical atherosclerosis [3]. Intensive glycemic control early in the course of the disease lowers cardiovascular events in the long term [4]. Despite all this evidence, very little is understood about the molecular mechanisms connecting hyperglycemia to atherosclerosis.

The nuclear factor of activated T-cells (NFATc1-c4) are a family of Ca^2+^/calcineurin-dependent transcription factors first characterized in T-lymphocytes as inducers of cytokine gene expression. Since then, NFAT proteins have been shown to play various roles outside immune cells, including in the cardiovascular system. We have previously shown that hyperglycemia effectively activates NFATc3 in the arterial wall [5], [6] and once activated, NFATc3 induces the expression of the pro-inflammatory matrix protein osteopontin (OPN), a cytokine that promotes atherosclerosis and diabetic vascular disease [6]. Diabetes increased OPN expression in the aorta of normolipidemic mice and this was prevented by pharmacological inhibition of NFAT with the NFAT-blocker A285222 or by lack of NFATc3 protein in NFATc3 deficient mice [6]. Additional experimental evidence supports a role for NFAT as a regulator of genes able to promote vascular dysfunction and potentially, a pro-atherogenic vascular phenotype [7], [8], [9]. NFAT promotes vascular smooth muscle cell (VSMC) proliferation and migration [7], [10], and plays a role in neointima formation and in the regulation of cyclooxygenase 2 (Cox2) expression after vascular injury [11], [12], [13]. NFAT contributes to the development of angiotensin II-induced hypertension, via down-regulation of potassium channel expression [14], [15]. Moreover, NFAT controls the alternative splicing of allograft inflammatory factor-1 (AIF-1), resulting in products differentially associated to parameters defining human plaque phenotype and stability [16].

Together, these observations led us to hypothesize that NFAT may act as a glucose-sensor in the vessel wall, translating changes in Ca^2+^ signals into changes in gene expression that lead to macrovascular disease in diabetes. To more directly test this hypothesis and in the context of an atherosclerosis-prone experimental model, we investigate the effects of NFAT-signaling inhibition on atherosclerotic plaque formation and inflammatory burden in diabetic and non-diabetic apolipoprotein (Apo)E deficient mice.

## Materials and Methods

### Animals

This study was carried out in strict accordance with the recommendations in the Guide for the Care and Use of Laboratory Animals of the National Institutes of Health. All protocols were approved by the local ethics review board at Lund University and the Malmö/Lund Animal Care and Use Committee (Permit Number: M29-12). Animals were anaesthetized with ketamine hydrochloride and xylazine (i.p.; 2.5 mg and 7.5 mg/100 g body weight, respectively) and euthanized by exsanguination through cardiac puncture for blood collection. Depth of anesthesia was assessed by the toe-pinch reflex procedure and absence of muscular tone. All efforts were made to minimize suffering. Adult ApoE^−/−^ (B6.129P2-Apoe*^tm1Unc^*/J; Charles River, Sulzfeld, Germany; n = 196), C57BL/6J (stock number 000664; Charles River; n = 25) and FVBN 9x-NFAT-luciferase reporter mice (NFAT-luc; n = 72) [17] were used.

### Study Design

**Figure 1:**
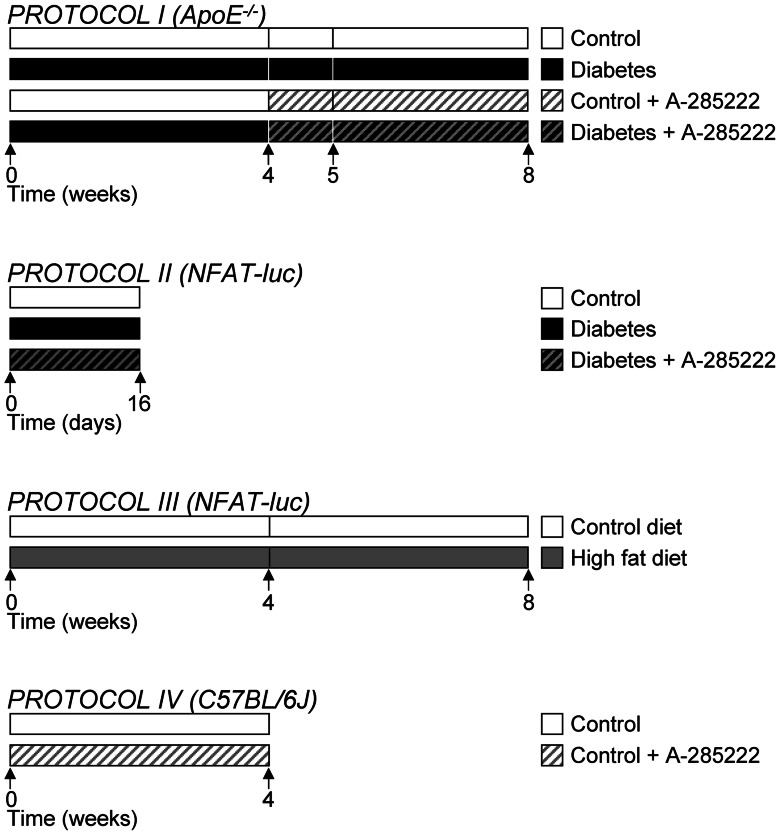
Study design. **Protocol I:** 22 weeks old ApoE^−/−^ mice received intraperitoneal (i.p.) injections of STZ or vehicle as previously described [6]. One group of mice was euthanized 4 weeks after the first STZ/vehicle injection, while additional 2 groups received daily i.p. injections of the NFAT blocker A-285222 (0.29 mg/kg body weight) or vehicle (saline) for 1 or 4 weeks until termination. **Protocols II** and **III:** NFAT-luc mice were used. In protocol II, diabetes was induced as in protocol I and mice received daily i.p. injections of A-285222 (0.15 mg/kg body weight) or saline until termination at day 16. In protocol III, mice were fed a normal chow or a high fat diet (HFD; 0.15% cholesterol, 21% fat) during 4 or 8 weeks. **Protocol IV:** C57BL/6J mice received daily i.p. injections of A-285222 (0.29 mg/kg body weight) or vehicle for 4 weeks until termination. Arrows indicate time of termination; diabetes (black bars), control (white bars), A-285222-treated (hatched bars) and high fat diet (grey bars).

Treatment protocols are summarized in Figure 1. **Protocol I**: 22 weeks old ApoE^−/−^ mice received intraperitoneal (i.p.) injections of streptozocin (STZ; Sigma-Aldrich, Stockholm, Sweden; 60 mg/kg body weight, pH 4.5) or vehicle (citrate buffer) once a day for 5 days at the start of the experiments, as previously described [6]. One group of mice (n = 24) was euthanized 4 weeks after the first STZ/vehicle injection, while additional 2 groups received daily i.p. injections of the NFAT blocker A-285222 (0.29 mg/kg body weight) or vehicle (saline) for 1 (n = 81) or 4 weeks (n = 43) until termination. **Protocols II** and **III:** NFAT-luc mice were used. In protocol II (n = 29), diabetes was induced as in protocol I and mice received daily i.p. injections of A-285222 (0.15 mg/kg body weight) or saline until termination at day 16. In protocol III (n = 36), mice were fed a normal chow (R3; Lantmännen, Kimstad, Sweden) or a high fat diet (HFD; R638∶0.15% cholesterol, 21% fat; Lantmännen) during 4 or 8 weeks. **Protocol IV**: C57BL/6J mice (n = 25) received daily i.p. injections of A-285222 (0.29 mg/kg body weight) or vehicle for 4 weeks until termination.

A-285222 inhibits all NFAT family members and was provided by Abbott Laboratories (Abbott Park, IL). Body weight and blood glucose, measured in whole venous blood (One-Touch, LifeScan Inc., Milipitas, CA) were monitored once a week. Animals had free access to tap water, fed normal chow diet (except in protocol III). For *en face* and cross sectional measurements of plaque (4- and 8-weeks groups), the aorta and heart were dissected out after whole body perfusion with phosphate-buffered saline (PBS) and stored in Histochoice (Amresco Inc, Solon, OH) at 4°C for fixation until further processing. For mRNA expression measurements in the aortic arch and experiments involving splenocytes and monocytes (5-weeks group), whole body perfusion with PBS was performed, after which aortas were dissected free of connective tissue and snap frozen, and whole spleens were weighed and stored in ice cold PBS until further processing. The 8-week version of protocol I was repeated in an additional group of diabetic mice (n = 25) for mRNA and protein expression measurements in the aortic arch and for liver histology. For the pharmacokinetics of A-285222, additional ApoE^−/−^ (n = 23) and FVBN (n = 7) mice were used.

### Histological Evaluation of Atherosclerosis, Spleen and Liver

*En face* preparations of the aorta were performed as described before [18]. Briefly, aortas were fixed in Histochoice, dipped in 78% methanol and stained for 40 min in 0.16% Oil Red O (ORO) dissolved in 78% methanol/0.2 mol/L NaOH, after which they were washed in 78% methanol and distilled water. Cover slips were mounted with water-soluble mounting media L-550A (Histolab, Göteborg, Sweden). Lipids are stained red, which makes the plaques bordeaux-colored. Lipid (ORO), macrophages (Moma-2; monocyte/macrophage 2), α-smooth muscle actin (α-SMA) and collagen contents were evaluated in cross-sections (10 µm) of the aortic root as described before [18]. Rat anti-Moma-2 primary antibody (1 µg/mL; BMA Biomedicals, Augst, Switzerland), mouse anti-alpha-SMA (0.42 µg/mL, Sigma-Aldrich) and biotinylated secondary IgG antibodies (Vector Laboratories, Burlingame, CA) were used. Sections were counter-stained with Harris hematoxylin for determination of subvalvular lesion area, expressed both in µm^2^ and as percentage of total cross sectional area to correct for potential structural differences in the arterial wall between groups [19]. Media and lumen areas were also determined based on the Harris hematoxylin staining. Specificity of immune staining was confirmed by the absence of staining when primary or secondary antibodies were omitted from the protocol. Sections (5–6 per mouse) were analyzed under blind conditions by computer-aided morphometry (Image-Pro Plus, Media Cybernetics, Bethesda, MD and BioPix iQ 2.0 software, Biopix AB, Gothenburg, Sweden, for *en face* and cross sections respectively). Expression of tissue factor (TF) and osteopontin (OPN) was also evaluated in the aortic root using confocal immunofluorescence microscopy as described before [6]. Sections were stained with primary rabbit antibodies, anti-OPN (0.5 µg/mL, IBL, Hamburg, Germany) or anti-TF (10 µg/mL, American Diagnostica, Stamford, CT) and secondary antibody, DyLight 649 anti-rabbit IgG (1∶400 and 1∶500, for OPN and TF, respectively; Jackson ImmunoResearch, West Grove, PA); and counterstained with the nucleic acid dye SYTOX Green (1∶3000, Molecular Probes, Invitrogen, Paisley, UK). Sections (3–6 per mouse) were examined under blind conditions at 20X in a Zeiss LSM 5 Pascal laser scanning confocal microscope and mean fluorescence intensities of OPN and TF in the plaque were quantified using the Zeiss LSM 5 analysis software and ImageJ (version 1.47 m), respectively.

Spleen and liver cryosections (10 µm) were fixed with Histochoice and stained with hematoxylin and eosin (H&E). Liver sections were also stained with ORO and hematoxylin. For quantification of liver fat content (ORO), three sections per mouse were analyzed under blind conditions by computer-aided morphometry (BioPix iQ 2.0 software, Biopix AB, Gothenburg, Sweden).

### Luciferase Reporter Assay

Luciferase activity was measured in tissue homogenates from the aortic arch, spleen, thymus, brain, heart, liver and kidney. Assays were performed as previously described [5], [7]. Optical density was measured using a Tecan Infinite M200 instrument (Tecan Nordic AB, Mölndal, Sweden) and data expressed as relative luciferase units (RLU) per µg protein. Protein concentration was determined with the EZQ protein quantification kit (Molecular Probes, Invitrogen, Paisley, UK) or the DC Protein Assay (Bio-Rad Laboratories Sundbyberg, Sweden).

### Plasma Cholesterol, Triglycerides and Cytokines

Plasma cholesterol and triglycerides were measured by colorimetric assays (Infinity™-Cholesterol and Infinity™-Triglyceride; Thermo Scientific, Middletown, VA) as described before [20]. Plasma cytokines were measured using a pro-inflammatory 7-plex kit (Meso Scale Discovery, Rockville, MD). The lower detection limit for each cytokine was within the range described by the manufacturer. Plasma OPN and soluble (s) vascular cell adhesion molecule 1 (VCAM-1) levels were assayed using Quantikine mouse OPN and sVCAM-1 ELISA kits (R&D Systems, Abingdon UK). Absorbance was measured at 450 nm and the lower limits of detection were 5.7 pg/mL and 0.31 ng/mL, respectively. All assays were performed according to the manufacturers’ instructions.

### Quantitative RT-PCR (qRT-PCR)

RNA was extracted from the aortic arch using TRI Reagent BD (Sigma-Aldrich) and a protocol for simultaneous isolation of RNA, DNA and protein, according to the manufacturer’s instructions. cDNA synthesis and real-time PCR were performed as previously described [21], using TaqMan Gene Expression assays for IL-6 (Mm00446190_m1), OPN (Mm00436767_m1), monocyte chemotactic protein 1 (MCP-1; Mm00441242_m1), intercellular adhesion molecule 1 (ICAM-1; Mm00516023_m1), VCAM-1 (Mm01320970_m1), IL-1β (Mm01336189_m1), Cox-2 (Mm00478374_m1), IL-10 (Mm00439614_m1), TF (Mm00436948_m1), CD68 (Mm03047340_m1) with HPRT (Mm00446968_m1) and β-actin (Mm00607939_s1) as endogenous controls.

### Western Blotting

Following RNA extraction from the aortic arch, protein was precipitated from the phenol-ethanol supernatant obtained after sedimentation of the DNA pellet. After a series of washes, the protein-containing pellet was dried and dissolved in SDS sample buffer (62.5 mmol/L Tris-HCl pH 6.8, 2% SDS, 10% glycerol, 5% 2-mercaptoethanol and 0.001% bromophenol blue). Alternatively, arteries were homogenized directly in SDS sample buffer as previously described [6]. Protein concentration was determined with the EZQ protein quantification kit (Molecular Probes). An equal amount of protein was loaded onto 12.5% Tris-HCl gels (Bio-Rad Laboratories) and separated by gel electrophoresis. Proteins were transferred to polyvinylidene difluoride membranes (Bio-Rad Laboratories), blocked in 3% BSA/5% non-fat dry milk and incubated with primary anti-TF (2 µg/mL in 3% BSA, American Diagnostica, Stamford, CT) or to nitrocellulose membranes (Bio-Rad Laboratories), blocked in 5% BSA and incubated with anti-CD68 (0.82 µg/mL in 5% milk, DakoCytomation, Glostrup, Denmark). HRP-conjugated secondary antibody (Cell Signaling, Danvers, MA) was used and bands detected with chemiluminescence (Supersignal West Dura, Pierce Biotechnology, Rockford, IL). β-actin (1∶3000) or α-tubulin (1∶5000; both from GenScript Corporation, Piscataway, NJ) were used as loading controls.

### Splenocyte Proliferation and Cytokine Production

Splenocytes were isolated as previously described [22]. Briefly, single cell suspensions were prepared by pressing spleens through a 70-µm cell strainer (BD Falcon, Franklin Lakes, NJ). Erythrocytes were removed using red blood cell lysing buffer (Sigma-Aldrich). Cells were cultured in culture medium containing 10% heat-inactivated FCS, 1 mmol/L sodium pyruvate, 10 mmol/L Hepes, 50 U of penicillin, 50 µg/mL streptomycin, 0.05 mmol/L β-mercaptoethanol, and 2 mmol/L L-glutamine (RPMI 1640, GIBCO, Paisley, UK) in 96-well round bottom plates (Sarstedt, Nümbrecht, Germany). For proliferation assay, 2×10^5^ cells/well were cultured with or without Dynabeads® coupled to anti-CD3 and anti-CD28 antibodies for T-cell activation and expansion (bead to cell ratio 1∶1, Invitrogen, Life Technologies, Carlsbad, CA); alternatively, with or without 2.5 µg/mL concanavalin A (ConA; Sigma-Aldrich) for 88 hours. To measure DNA synthesis, the cells were pulsed with 1 µCi [methyl-^3^H]thymidine (Amersham Biosciences, Uppsala, Sweden); during the last 16 hours, macromolecular material was harvested on glass fiber filters using a Filter Mate Harvester (Perkin Elmer, Buckinghamshire, UK) and analyzed using a liquid scintillation counter (Wallac 1450 MicroBeta, Ramsey, MN). For cytokine production, 1×10^6^ cells/well were cultured in 48-well plates (Sarstedt, Nümbrecht, Germany) with or without anti-CD3/CD28 dynabeads (bead to cell ratio 1∶1, Invitrogen) for 48 hours, after which cytokine concentrations in the culture media were measured using a Th1/Th2 9-plex ultra-sensitive kit (Meso Scale Discovery). The lower detection limit for each cytokine was within the range described by the manufacturer.

### Flow Cytometry Analysis

Cells from blood and splenocytes were analyzed as previously described [23], [24], [25]. Blood cells were stained with the following fluorochrome-conjugated antibodies after blocking of FC receptors for 5 minutes: PerCP/Cy5.5-anti-CD62L (L-selectin), PE/Cy7-anti-Ly-6c, (BioLegend, San Diego, CA) and APC-anti-CD115 (eBioscience, San Diego, CA). Splenocytes were stained for PE/Cy7-anti-CD3, Pacific Blue-anti-CD4, APC-anti-CD25 after blocking of FC receptors for 5 minutes. Cells where then permeabilized and thereafter stained with PE-anti-Foxp3 (Biolegend). For interferon (IFN)-γ measurements, splenocytes (5×10^5^ cells/cell culture well) were incubated with phorbol 12-myristate 13-acetate (PMA; 10 ng), ionomycin (0.2 µg), and brefeldin A (1 µg, all from Sigma) for 4 hours at 37°C. Stimulated cells were then stained for CD3 and CD4 (as above). Cells were thereafter permeabilized and stained with PE-anti-IFN-γ (Biolegend). Measurements were performed using a CyAn ADP flow cytometer (Beckman Coulter, Brea, CA) and analyzed with FlowJo7.6 software (Tree Star, Ashland, OR). Mononuclear leukocytes were gated from the forward scatter (FSC)/side scatter (SSC). Single stained samples were used to correct for fluorescence spillover in multicolor analyses, and gate boundaries were set by fluorescence-minus-one (FMO) controls.

### Gas Chromatography Mass Spectroscopy (GC/MS)

For the pharmacokinetics of A-285222, blood was collected from adult ApoE^−/−^ mice (n = 23) by cardiac puncture at different time points (30 min, 1, 2, 4, 6, 12 and 24 hours) after i.p. injection of A-285222 (0.29 mg/kg body weight in saline solution). Plasma was isolated and a known concentration (2.5 µmol/L) of the analogous inactive compound A-216491 (Abbott Park, IL) was added as an internal standard to all samples. Samples were randomized and run in duplicates. Samples (300 µL) were extracted twice with ethyl acetate (400 µL), followed by evaporation. The dried residues were finally re-dissolved in chloroform (30 µL) and analyzed by GC/MS on an Agilent 6890N gas chromatograph (Agilent, Santa Clara, CA) coupled to a Leco Pegasus III TOFMS electron impact TOF (time-of-flight) mass spectrometer (Leco Corp., St. Joseph, MI). Identification was based on mass spectra and retention indexes, calculated from the injection of a homologous series of n-alkanes. The concentration of A-285222 in plasma was determined using a calibration curve calculated from analyses of plasma from untreated mice, spiked with known concentrations of A-285222 and A-216491. Plasma A-285222 levels were also determined in mice from protocols I (5 and 8 weeks), II and IV, from blood collected at the time of euthanasia (i.e.∼24 h after the last i.p. injection of A-285222). These measurements were performed in duplicate using pooled plasma from 6–12 mice for each experimental condition. Plasma from the groups treated with saline served as negative controls. In a separate experiment to evaluate efficacy of i.p. administration, plasma levels of A-285222 after intracardiac (i.c.) injection of the drug were compared to levels after i.p. administration (n = 7 mice).

### Statistics

Results are expressed as means ± SEM if not otherwise specified. Statistical analysis was performed using GraphPad software (Prism 5.0). For parametric data, significance was determined using Student’s t-test, one- or two-way ANOVA as specified in the text, followed by Bonferroni post hoc tests. Non-parametric data was analyzed using Mann-Whitney or Kruskal-Wallis test followed by Dunn’s post-test.

## Results

### *In vivo* Inhibition of NFAT Prevents the Diabetes-induced Aggravation of Atherosclerosis in the Aortic Arch

**Figure 2:**
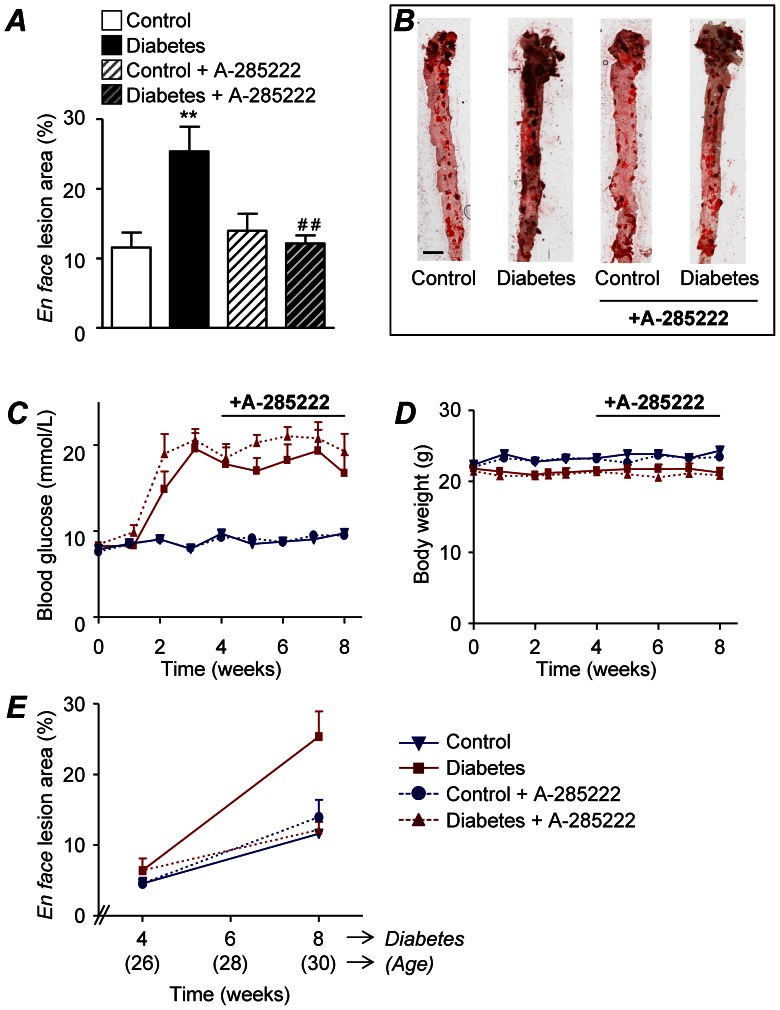
NFAT inhibition suppresses accelerated atherosclerosis in diabetes. (**A**) *En face* lesion area in the aortic arch of control and diabetic female ApoE^−/−^ mice treated for 4 weeks with A-285222 or saline (protocol I). Mice were 30 weeks old at the time of analysis, 8 weeks after the first STZ or vehicle injection. Data is expressed as percentage of total aortic arch area (n = 9–12 mice/group). Two-way ANOVA for the effect of diabetes and the drug revealed significant interaction between factors (**P<0.005). Bonferroni post-test yielded ^**^P<0.01 vs. non-diabetic control mice and *^##^P<0.01* vs. diabetic saline-treated mice. (**B**) Representative *en face* preparations of aortas from ApoE^−/−^ mice treated as in A and stained with ORO (bordeaux-colored). Scale = 2 mm. (**C**) Blood glucose (mmol/L) and (**D**) body weight (g) values for mice in panel A. (**E**) Merged data from the measurements in panel A and *en face* data obtained 4 weeks after the first STZ or vehicle injection (n = 9–13 mice/group). Control non-diabetic (blue); diabetic (red); A-285222-treated (dotted lines); saline treated (unbroken lines).

Atherosclerosis prone 22 week old ApoE^−/−^ mice were treated as outlined in PROTOCOL I (Figure 1). A 2.2 fold increase in atherosclerotic plaque area (25.4% vs. 11.6%; p<0.01) was observed in the aortic arch of diabetic mice when compared to aged-matched non-diabetic controls, as assessed by *en face* measurements of ORO stained area eight weeks after the first STZ-injection (Figure 2A–B). *In vivo* treatment with the NFAT blocker A-285222 for the last 4 weeks of the experiment completely abrogated (p<0.01) the effect of diabetes on lesion area (Figure 2A–B). There was no effect of A-285222 on atherosclerosis in non-diabetic ApoE^−/−^ mice. As expected, STZ-treated mice had higher blood glucose and lower weight gain than control mice, but A-285222 had no impact on these parameters (Figure 2C–D and Table 1). A similar effect of diabetes was observed in the descending aorta (2.1 fold increase in plaque area), but the overall plaque area was lower than in the arch and the effect of A-285222 less pronounced (Figure S1).

**Table 1:** Blood glucose, body weight, plasma cholesterol and triglyceride values in mice undergoing the different experimental protocols.

Genotype and treatment	Blood glucose(mmol/L)	Body weight (g)	Total cholesterol(mmol/L)	Triglycerides(mmol/L)
**Protocol I (ApoE^−/−^)**				
***4 weeks:***				
Control (n = 13)	8.8±0.8	22.9±1.4	10.8±1.5	0.48±0.13
Diabetes (n = 11)	19.6±4.7***	21.6±1.9	16.9±3.5***	0.55±0.22
***5 weeks:***				
Control (n = 9)	9.1±1.1	30.2±2.0	10.6±1.4	1.44±0.36
Diabetes (n = 11)	23.4±5.5***	29.4±1.7	17.9±2.4**	2.71±1.02
Control+A-285222 (n = 8)	9.6±0.5	30.6±2.1	13.6±3.2	2.14±1.03
Diabetes+A-285222 (n = 11)	23.2±5.8***	28.3±2.6*	17.2±5.6	2.57±1.56
***8 weeks:***				
Control (n = 12)	9.0±1.6	24.3±1.7	10.2±1.3	0.65±0.22
Diabetes (n = 10)	17.7±5.5***	21.3±2.2***	15.6±4.4***	1.04±0.38**
Control+A-285222 (n = 12)	9.0±0.4	23.4±1.3	11.7±1.4	0.68±0.19
Diabetes+A-285222 (n = 9)	19.8±3.5***	20.8±1.0**	16.0±4.7**	0.93±0.25
**Protocol II (NFAT-luc)**				
Control (n = 11)	8.9±1.0	22.5±3.0	2.30±0.33	1.74±0.39
Diabetes (n = 8)	15.4±7.2*	21.9±1.4	2.49±0.33	2.20±0.30
Diabetes+A-285222 (n = 10)	14.7±5.4*	22.7±2.3	2.45±0.36	1.91±0.75
**Protocol III (NFAT-luc)**				
***4 weeks:***				
Control (n = 12)	10.3±1.2	30.8±4.3	3.19±0.34	1.31±0.56
High fat diet (n = 8)	11.4±1.6	39.0±7.7**	5.59±1.25***	0.80±0.38
***8 weeks:***				
Control (n = 9)	9.7±0.7	35.9±5.6	3.39±0.67	1.24±0.53
High fat diet (n = 7)	12.7±3.0**	43.7±5.0*	6.00±1.01***	1.04±0.40
**Protocol IV (C57Bl6/J)**				
Control (n = 13)	12.8±2.7	25.2±3.4	2.75±0.73	0.50±0.13
Control+A-285222 (n = 12)	11.7±1.9	26.0±4.5	2.42±0.46	0.49±0.15

Values represent mean ± SD. Blood glucose values are averaged during the experiments (from week 2 until termination). Body weight and lipids values were measured at termination. For protocol 1 (4 weeks), student’s t-test yielded ***p<0.001 vs. non-diabetic mice. For protocol 1 (5 and 8 weeks), two-way ANOVA (for the effects of diabetes and A-285222) revealed no interactions between factors. Bonferroni post-tests yielded *<0.05, **p<0.01, ***p<0.001 for comparisons between control and diabetic mice receiving the same treatment. For protocol 2, one-way ANOVA and Bonferroni post-tests yielded *<0.05, vs non-diabetic control. For protocol 3, two-way ANOVA (for the effects of high fat diet and diet duration) revealed no interactions. Bonferroni post-tests yielded *<0.05, **p<0.01, ***p<0.001 for comparisons between mice fed high fat diet and controls.

Lesion area was also evaluated in a separate group of control and diabetic mice 4 weeks after the first STZ or vehicle injection, when mice were 26 weeks of age. At this earlier time point diabetes had no evident effect on lesion size in the aortic arch (Figure 2E). Figure 2E also shows the accelerated development of plaque in the diabetic mice, which is completely prevented by NFAT inhibition, and the well-recognized effect of age on atherosclerosis [26] in the non-diabetic groups.

**Figure 3:**
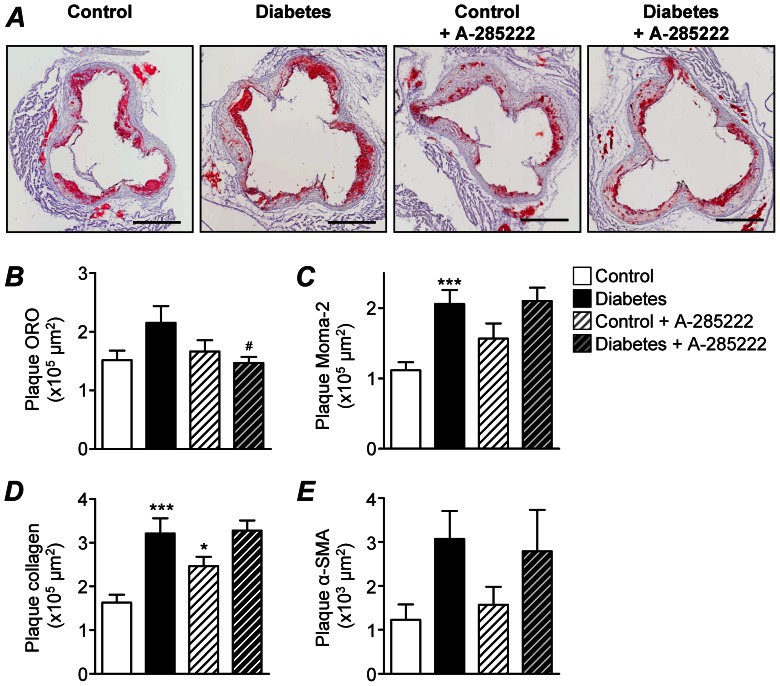
Inhibition of NFAT reduces the lipid contents in the plaque of diabetic mice. (**A**) ORO stained cross-sections of the aortic root from control and diabetic female ApoE^−/−^ mice treated with or without A-285222 for 4 weeks (Protocol I). Sections were counter-stained with Harris hematoxylin. Scale = 500 µm. (**B**) Summarized morphometric data from sections stained as in A showing ORO positive area in the plaques. Two-way ANOVA for the effect of diabetes and the drug revealed significant interaction between factors (*P<0.05*). Post-test yielded *^#^P<0.05* vs. diabetic saline-treated mice (n = 9–12 mice/group). (**C–E**) Summarized morphometric data from the same animals as in B showing monocytes/macrophages-2 (Moma-2, C), collagen (D) and α-smooth muscle actin (α-SMA, E) positive areas in the plaques. Two-way ANOVA revealed significant effect of diabetes (*P<0.001* for Moma-2 and collagen; *P<0.05* for α-SMA), Bonferroni post-test yielded ^*^P<0.05 and ^***^P<0.001 vs. non-diabetic saline-treated mice (n = 9–12 mice/group).

Atherosclerosis was also examined in cross-sections of the aortic root from the same animals used for the *en face* measurements shown in Figure 2A–B. Total cross sectional area calculated from the hematoxylin staining was larger in diabetic than non-diabetic mice (1.68×10^6^ um^2^ vs. 1.23×10^6^ um^2^; P<0.001). This increased area in the diabetic mice is the combined result of increased plaque (6.59×10^5^ um^2^ vs. 4.03×10^5^ um^2^; P<0.001) and lumen (9.04×10^5^ um^2^ vs. 6.65×10^5^ um^2^; P<0.001) area, while a concomitant thinning of the media was observed (1.15×10^5^ um^2^ vs. 1.83×10^5^ um^2^; P<0.001), especially in regions beneath the atherosclerotic plaques. Erosion and focal dilatation of the medial layer have been described in the aortic root of ApoE^−/−^ mice when fed an atherogenic diet with cholate or when bred on specific backgrounds (C3H or 128SvJ) [27], [28]. Our results show that diabetes (without the atherogenic diet or specific background) is a sufficient drive for medial wall erosion; in line with work showing structural modifications and disruption of the arterial media as a result of diabetes in STZ-treated C56B/J6 mice [19]. When the subvalvular plaque area was expressed as percentage of total cross sectional area, it was 16.5% larger in diabetic than in control untreated mice (p<0.05); whereas the effect of diabetes was attenuated in A-285222-treated mice (9.6% increase, n.s.). Interestingly, inhibition of NFAT resulted in reduced lipid contents in the plaque of diabetic mice (Figure 3A–B). Even though plaque macrophage, collagen and α-SMA contents were increased in the aortic root of diabetic mice when compared to non-diabetic controls, values were not affected by A-285222 treatment (Figure 3C–E and Figure S2A). Higher expression of OPN, but not of TF was observed in aortic root sections from diabetic mice when compared to non-diabetic controls; but values were not affected by A-285222 treatment either (Figure S2A–B).

In agreement with previous studies [20], total plasma cholesterol was significantly elevated in diabetic ApoE^−/−^ mice when compared to non-diabetic controls at 4, 5 and 8 weeks (Table 1). Plasma triglycerides were also elevated after 8 weeks. However, the reduced atherosclerosis is less likely due to a lipid lowering effect, since A-285222 had no effect on plasma lipids, regardless if the mice were diabetic or not (Table 1). In agreement to what others have reported [29], ApoE^−/−^ mice on chow diet had very little fat accumulation in the liver (<2%). No signs of liver steatosis were observed after treatment of ApoE^−/−^ mice with A-285222 (Figure S3).

### *In vivo* Treatment with A-285222 Effectively Blocks Diabetes-induced NFAT-dependent Transcriptional Activity

**Figure 4:**
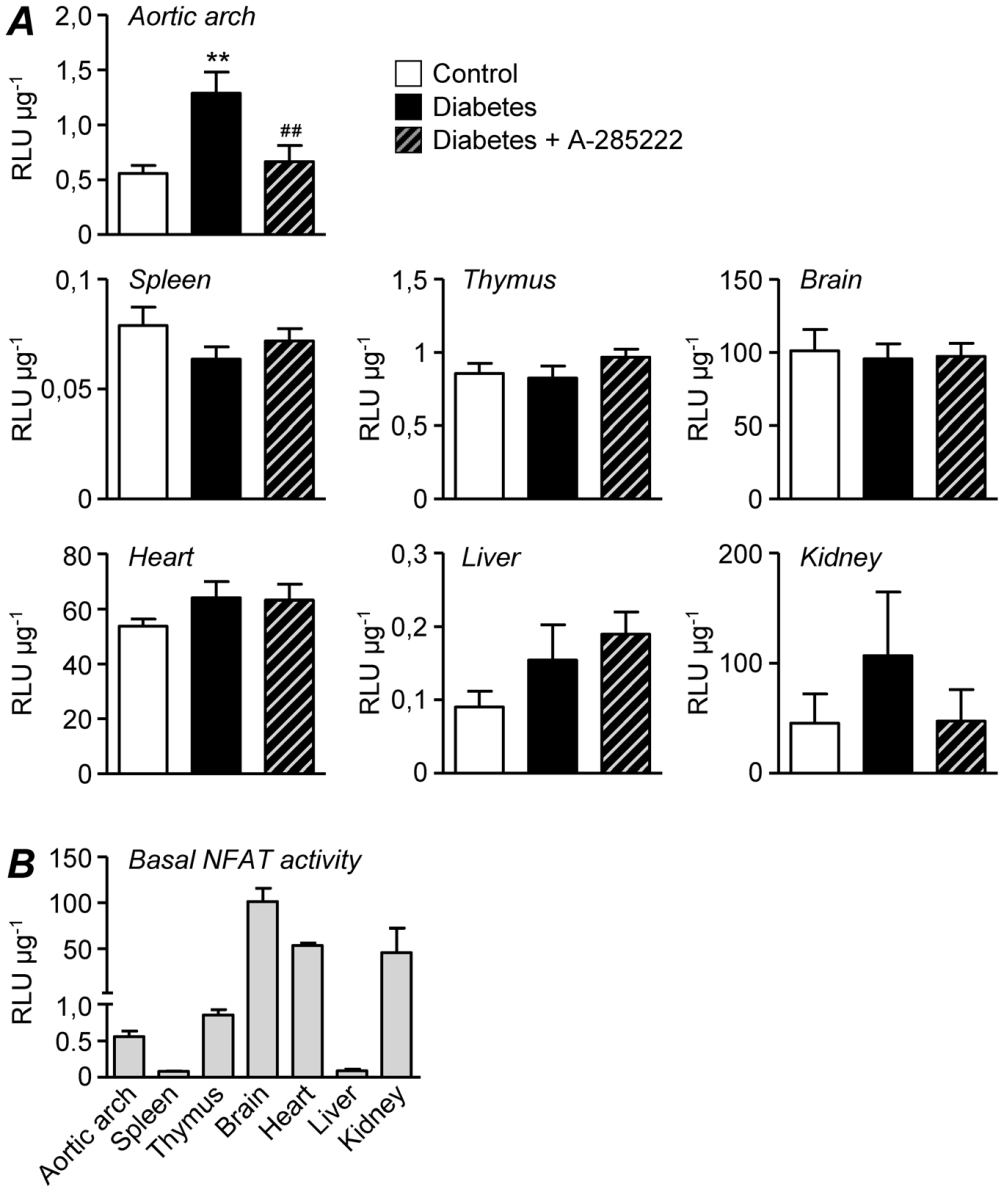
*In vivo* treatment with A-285222 effectively blocks diabetes-induced NFAT-dependent transcriptional activity. (**A**) NFAT-luciferase activity in the aortic arch, spleen, thymus, brain, heart, liver and kidney from control and diabetic female NFAT-luc mice treated with A-285222 or saline (Protocol II). Values are expressed as relative luciferase units (RLU) per µg protein (n = 8–11 mice/group for aortic arch, spleen and thymus; n = 4–8 mice/group for brain, heart, liver and kidney). ^**^P<0.01 vs. non-diabetic control mice and *^##^P<0.01* vs. diabetic saline-treated group. (**B**) Basal (non-diabetic) NFAT-dependent transcriptional activity (RLU µg^−1^) in the different tissues examined (n = 4–11 mice/group).

Previous studies from our group have shown that A-285222 is a potent blocker of glucose-induced NFAT-dependent transcriptional activity and NFAT-target genes [6], [7] in VSMCs and intact vessels *in vitro* [5], [6]. Here we demonstrate that *in vivo* treatment with A-285222 completely blocks the diabetes-induced NFAT-dependent transcriptional activity in the aortic arch of NFAT-luc mice (Figure 4A, PROTOCOL II). Of all organs examined, NFAT was selectively activated in the aorta of diabetic mice, with a tendency to increased activation in kidneys, but no effect in spleen, thymus, brain, heart or liver. A-285222 treatment was effective only if NFAT had been previously activated as in the aortic arch, and possibly in the kidneys. Diabetic mice had significant hyperglycemia at this time-point, but unchanged body weight and plasma lipids (Table 1). A-285222 had no effect on any of these parameters (Table 1). As shown in Figure 4B, basal levels of NFAT activity varied depending on the organ. Consistent with what others have reported, the highest levels were observed in brain, kidney and heart; and the lowest in spleen and liver [17].

A separate set of NFAT-luc mice were fed HFD or chow diet for 4 or 8 weeks and NFAT-luciferase activity measured in the aorta (PROTOCOL III). Even though plasma cholesterol levels were significantly higher in mice fed HFD when compared to controls (Table 1), no differences in NFAT-luciferase activity were observed (Figure S4). Body weight increased with HFD, and a small (3 mmol/L) but significant increase in blood glucose was observed after 8 weeks of HFD, while triglycerides were not significantly affected (Table 1).

### Effect of Diabetes and NFAT-signaling Inhibition on Systemic and Plaque Inflammation

**Figure 5:**
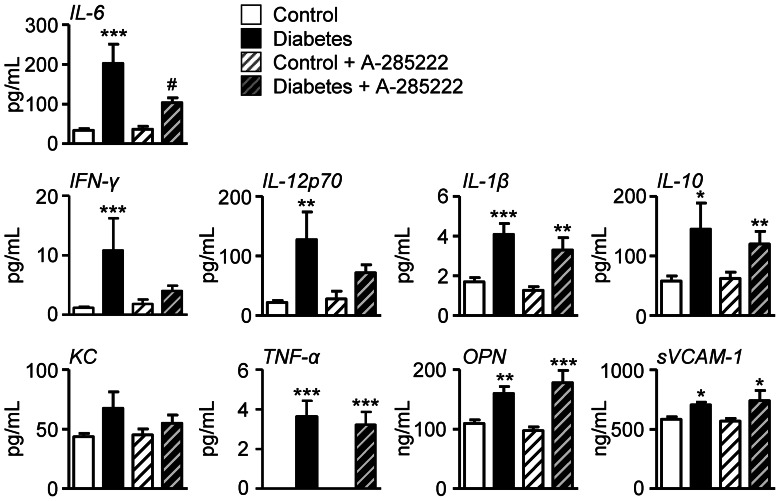
*In vivo* NFAT inhibition reduces diabetes-induced elevation of plasma IL-6 levels. The effect of diabetes and NFAT inhibition on plasma IL-6, IFN-γ, IL-12p70, IL-1β, IL-10, KC, TNF-α, OPN and sVCAM-1 was studied 8 weeks after the first STZ/vehicle injection in female ApoE^−/−^ mice treated with A-285222 or saline for the last 4 weeks of the experiment (Protocol I, n = 9–12 mice/group). Only for IL-6, two-way ANOVA for the effect of diabetes and the drug revealed a significant interaction between factors (*P<0.05). Bonferroni post-test yielded ^***^P<0.001 vs. non-diabetic control and *^#^P<0.05* vs. diabetic saline-treated group. All other cytokines except KC were increased by diabetes. For parametrically distributed data (IL-1β and OPN), two-way ANOVA followed by Bonferroni post-test yielded ^**^P<0.01 and ^***^P<0.01 vs. corresponding non-diabetic groups. For the rest of the cytokines, which were non-parametrically distributed, Kruskal-Wallis followed by Dunn’s post-test yielded ^*^P<0.05, ^**^P<0.01 and ^***^P<0.01 vs. corresponding non-diabetic groups.

**Figure 6:**
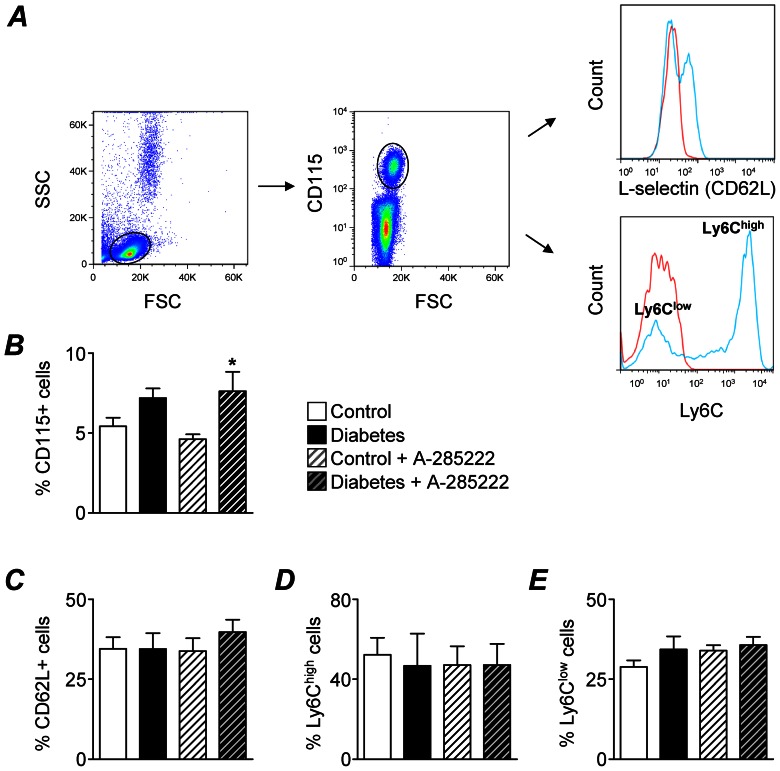
Circulating monocyte levels are increased in diabetic ApoE^−/−^ mice but not affected by NFAT inhibition. (**A**) Left and middle panels: Dot plots showing the gating strategy for monocyte identification from blood mononuclear cells based on the expression of CD115 (M-CSF receptor). Right panels: Identification of monocytes expressing CD62L (L-selectin) and of Ly6C^high^ and Ly6C^low^ subsets (blue lines); fluorescence-minus-one controls (red lines). (**B**) Summarized data from flow cytometry experiments showing percentages of CD115+ monocytes in total blood mononuclear cells from control and diabetic ApoE^−/−^ mice treated with the NFAT blocker A-285222 or saline for one week (Protocol I, n = 9–11 mice/group). Two-way ANOVA revealed a significant effect of diabetes (*P<0.01*). Bonferroni post-test yielded ^*^P<0.05 vs. non-diabetic control mice. (**C–E**) No differences in the percentages of CD115+ mononuclear cells expressing CD62L+ (C) Ly6C^high^ (D) or Ly6C^low^ (E) were found between treatment groups.

Plasma cytokines, levels and phenotype of circulating monocytes and expression of inflammatory genes in the aortic arch were examined in ApoE^−/−^ mice undergoing PROTOCOL I. Plasma IL-6, IFN-γ, IL-12p70, IL-1β, IL-10, tumor necrosis factor (TNF)α, OPN and sVCAM-1 were significantly increased in diabetic mice 8 weeks after the first STZ injection, whereas keratinocyte-derived chemokine (KC) was not affected (Figure 5). Treatment with A-285222 for 4 weeks blunted the effect of diabetes on IL-6 levels, but had no significant effects on the other plasma cytokines (Figure 5). Already early after the onset of diabetes, circulating monocytes were elevated in diabetic ApoE^−/−^ mice when compared to non-diabetic controls, as evidenced by higher percentages of CD115 (M-CSF receptor) expressing blood mononuclear cells (Figure 6B). Treatment with A-285222 had no effect on blood monocyte levels. Neither diabetes nor NFAT inhibition had any impact on the fraction of CD115 positive cells expressing the adhesion molecule CD62L (L-selectin) or on the fractions of inflammatory (CD115+ Ly6C^high^) or patrolling (CD115+ Ly6C^low^) blood monocytes (Figure 6C–E).

**Figure 7:**
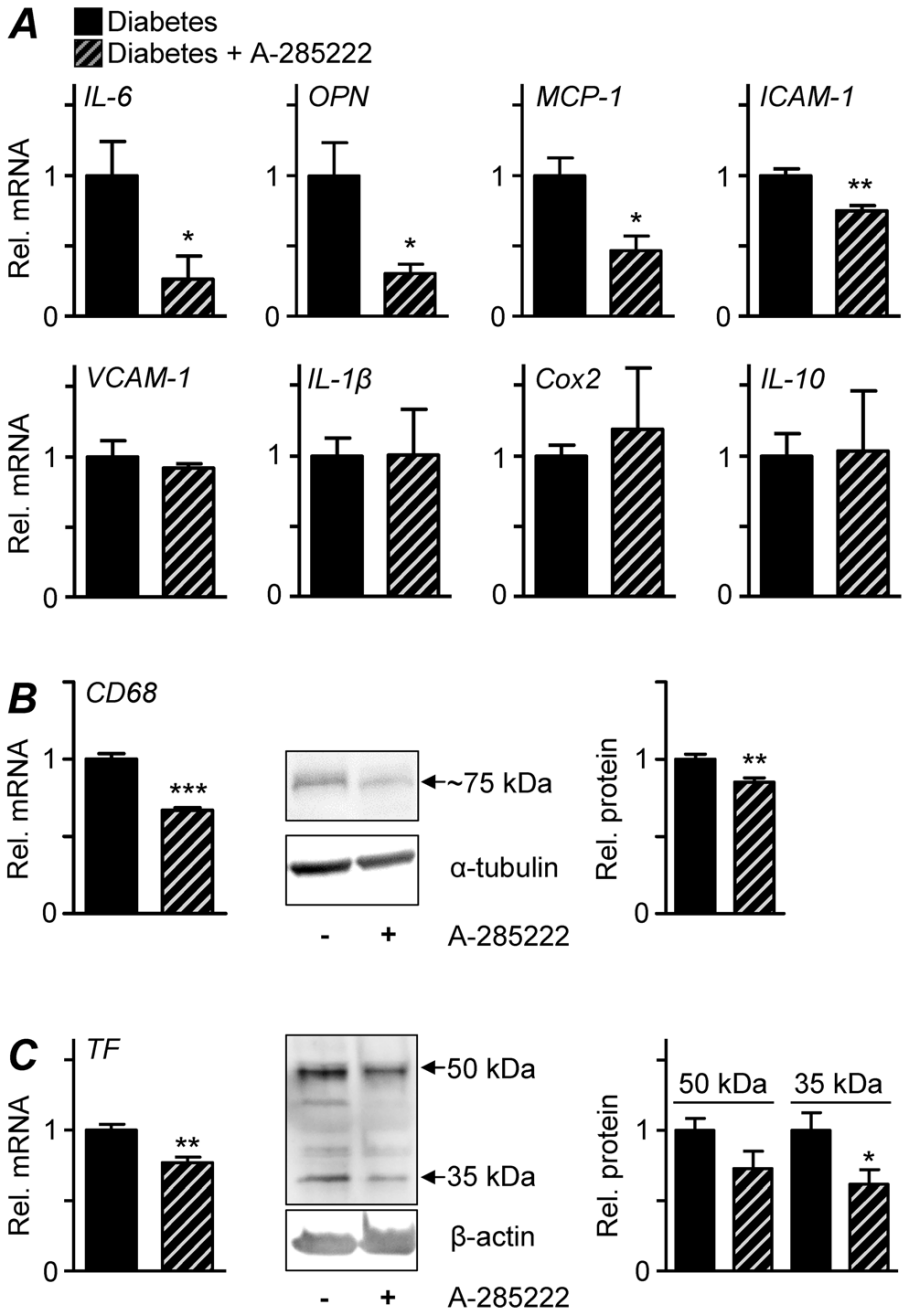
*In vivo* inhibition of NFAT reduces the expression of markers of inflammation and endothelial activation and macrophage infiltration in the aortic arch of diabetic mice. (**A**) Gene expression analyses by qRT-PCR in the aortic arch of diabetic ApoE^−/−^ mice treated with A-285222 or saline for 4 weeks (Protocol I, hatched and black bars, respectively). HPRT and β-actin were used as endogenous controls and data (Rel. mRNA) is expressed in relation to the diabetic saline-treated group. (**B**) CD68 and (**C**) TF mRNA and protein levels were decreased after treatment with A-285222. Left graphs show relative CD68 and TF mRNA from the same animals as in A. Middle panels show representative immunoblots for CD68 and TF (50 and 35 kDa) and loading controls. Right graphs show summarized results from western blot experiments, with CD68 expression normalized to α-tubulin and TF expression normalized to β-actin. Data (Rel. protein) is expressed in relation to the diabetic saline treated group. ^*^P<0.05, ^**^P<0.01 and ^***^P<0.001; n = 5–13 mice/group.

Also early after the onset of diabetes, several markers of inflammation and endothelial activation including MCP-1, VCAM-1, IL-1β, Cox2 and TF were significantly increased at the mRNA level in the aortic wall of diabetic mice when compared to controls; and trends towards increased IL-6 and ICAM-1 were observed (Figure S5). Treatment with A-285222 for 4 weeks significantly reduced IL-6, OPN, MCP-1 and ICAM-1 mRNA in the aortic arch of diabetic mice, while levels of VCAM-1, IL-1β, Cox2 and IL-10 were not affected (Figure 7A). Expression of the macrophage marker CD68 and cellular TF were significantly lowered after A-285222 treatment in diabetic mice, both at the mRNA and protein level (Figure 7B–C).

To determine whether NFAT signaling inhibition affected the capacity of immune cells to proliferate, splenocytes were isolated from ApoE^−/−^ mice 5 weeks after the first STZ injection, after receiving daily i.p. injections of A-285222 or vehicle for one week (PROTOCOL I, 5 weeks group). No differences in their proliferative capacity were found between groups, as assessed by measurements of thymidine incorporation under non-stimulated conditions or after stimulation with either anti-CD3/CD28 beads or Con A (Figure S6A–B). Similar observations were made using splenocytes isolated from control C57BL/6 mice treated with A-285222 for 4 weeks (Figure S6C). Blood glucose, body weight and plasma lipids were not affected in these animals (Table 1). We also examined the effects of diabetes and A-285222 treatment on cytokine secretion capacity of splenocytes under control non-stimulated conditions and after stimulation with anti-CD3/CD28 beads (Table S1). Diabetes had no impact on the levels of cytokines produced by non-stimulated splenocytes, but resulted in significantly increased levels of TNF-α in cells stimulated with anti-CD3/CD28 beads. A-285222 treatment on the other hand resulted in decreased secretion of IL-2 in non-stimulated splenocytes from non-diabetic mice and no effects in stimulated splenocytes (Table S1). Percentages of CD3+CD4+CD8- splenocytes expressing IFN-γ after stimulation with PMA and ionomycin were not affected by diabetes or NFAT inhibition (Figure S6E). Moreover, no differences in spleen histology or size were detected between treatment groups (Figure S6F–G).

### Pharmacokinetics of A-285222

Previous studies in cynomolgus monkeys have demonstrated that A-285222 is well tolerated when the plasma concentration is maintained below 4 µg/mL (9.6 µmol/L), a level achieved by oral administration of the drug twice daily at 5–7.5 mg/kg body weight [30]. In our hands, a lower dose was sufficient for changes in vascular OPN expression in normolipidemic BalB/c mice [6] and for the effects on NFAT-transcriptional activity and on diabetes-induced atherosclerosis described here (0.15–0.29 mg/kg body weight). In previous *ex vivo* experiments using mouse arteries, A-285222 blocked NFAT-transcriptional activity at 1 µmol/L [5]. To assess the actual plasma concentration of A-285222 after *in vivo* treatment of ApoE^−/−^ mice, we collected blood by cardiac puncture at different time points (30 min, 1, 2, 4, 6, 12 and 24 hours) after i.p. injection of the drug (0.29 mg/kg body weight). A-285222 was identified and quantified with GC/MS, based on its mass spectra and retention indexes (Figure S5). Plasma A-285222 levels peaked at 2 hours, were between 100–200 nmol/L for the first six hours and no longer detected at 12 or 24 hours. We failed to detect any A-285222 in mice from protocols I (5 and 8 weeks), II and IV, from blood collected at the time of euthanasia (i.e.∼24 h after the last i.p. injection of A-285222), ruling out an accumulation of the drug in the circulation. A comparison between plasma levels of A-285222 measured 5 min after i.p. injection of the drug (0.15 and 1.5 mg/kg) and after direct injection into the circulation (i.c.), showed that levels were within the same range regardless administration route (430 nmol/L vs. 480 nmol/L for the low dose; 770 nmol/L vs. 675 nmol/L for the high dose), indicating high bioavailability.

## Discussion

The present study demonstrates that inhibition of NFAT-signaling completely suppresses accelerated atherosclerosis in the aortic arch of diabetic ApoE^−/−^ mice and that this effect is independent of changes in plasma glucose or lipid levels. This finding suggests that NFAT may play a role in the development of atherosclerosis in diabetes and identifies this signaling pathway as a novel therapeutic target for the treatment of diabetic macrovascular complications.

The ApoE-deficient mouse is a well-established model for the study of atherosclerosis. Mice develop spontaneous hypercholesterolemia and mimic the initial phases of human atherosclerosis, even when fed a regular chow diet as in this study. The extent and severity of the lesions increase with age, displaying all known phases of atherogenesis. Monocyte adhesion takes place between 8–10 weeks of age, lipid deposition and development of fatty-streaks starts at approximately 9 weeks of age, and progression to intermediate and more mature fibrous plaques at ∼15–20 weeks of age [31]. As shown in Figure 2E, at the age when mice were treated with the NFAT blocker A-285222 (26–30 weeks of age), plaque size in the aortic arch was still increasing, and this was clearly accelerated by diabetes. Interestingly, NFAT inhibition did only affect the diabetes-driven aggravation of atherosclerosis, but had no impact on atherosclerosis in non-diabetic mice, suggesting potentially different mechanisms underlying plaque formation under diabetic and non-diabetic conditions. The increased lesion size observed in the aortic arch is in line with what others have described in STZ-treated ApoE^−/−^ mice [32]. However, the effect of diabetes on plaque area at the level of the aortic root was modest when compared to those observed when mice are treated with STZ at younger age (6 weeks [32] vs. 22 weeks in this study). Along these lines, A-285222 treatment had a more distinct impact on the aortic arch than in the aortic root, as exemplified by decreased macrophage infiltration, TF and OPN expression in the arch (Figure 7) but not in the root of the aorta (Figure S2). These results highlight differential susceptibility to diabetes-induced atherosclerosis in these two segments of the aorta and the need for a diabetes-driven process for NFAT-inhibition to play a role.

Previous work from our group established that high extracellular glucose (>15 mmol/L) activates NFATc3 in intact arteries *ex vivo* by a mechanism involving the release of extracellular nucleotides (i.e. UTP, UDP) acting on P2Y receptors, leading to increased intracellular Ca^2+^ and subsequent activation of the calcineurin/NFATc3 signaling pathway [5]. High glucose also decreases the export of NFATc3 from the nucleus by inhibiting the otherwise constitutively elevated kinase activity of glycogen synthase kinase (GSK)-3β and c-Jun N-terminal kinase in the arterial wall [5]. In a follow-up study, we demonstrated that hyperglycemia readily activated NFATc3 in the arterial wall *in vivo*, as evidenced by increased NFATc3 nuclear accumulation in cerebral arteries after an i.p. glucose-tolerance test and by increased NFATc3-dependent transcriptional activity in aorta 2 weeks after the induction of diabetes with STZ [6]. Here we show that this diabetes-induced activation of NFAT in the aorta is completely inhibited by *in vivo* treatment with A-285222 (Figure 4A), demonstrating that A-285222 is an effective blocker of NFAT-transcriptional activity in this tissue. Even though NFAT is expressed in many tissues and basal (non-diabetic) NFAT-luciferase activity was detected in all tissues examined, the diabetes-induced NFAT activation is not a generalized phenomenon. At least at this time point after the onset of diabetes (2 weeks), NFAT-luciferase activity seemed only elevated in the aorta, whereas no changes were observed in the other organs examined (Figure 4), an advantageous difference from the therapeutic point of view.

Not only hyperglycemia, but hyperlipidemia, or the combination of both could be driving the accelerated atherosclerosis in diabetes. To our knowledge, the effect of hyperlipidemia on NFAT-transcriptional activity in the vasculature has never been studied *in vivo*. A number of *in vitro* studies though, demonstrated that NFAT activation can be triggered by lipids. Exposure to triglyceride-rich very low-density lipoproteins increases NFATc3 nuclear accumulation in cultured rat aortic VSMCs [33], and postprandial triglyceride-rich lipoproteins collected after an oral fat load activate several transcription factors including NFAT in cultured endothelial cells [34]. Also, incubation of T-lymphocytes, macrophages, fibroblasts and endothelial cells with copper-oxidized or monocyte-oxidized low-density lipoproteins increases NFAT binding to DNA [35], [36]. Here we show that a ∼2-fold increase in total cholesterol induced by HFD, had no effect on NFAT-transcriptional activity in the aortas of NFAT-luc mice (Figure S4). Interestingly, after 8 weeks of HFD mice had a mild but still significant increase in blood glucose (from 9.7 to 12.7 mmol/L; Table 1), which did not translate in enhanced luciferase activity. This is in line with previous data showing that glucose levels >15 mmol/L are required for NFAT activation in the vasculature [5], [6]. Even though high cholesterol *per se* had no effect on NFAT-transcriptional activity *in vivo*, it is still possible that high triglycerides instead, or even higher absolute levels of cholesterol (such as those observed in ApoE-deficient mice), or higher degree of lipid oxidation as it may occur in the context of diabetes, could trigger NFAT activation.

Inflammation is recognized as a critical regulator of atherosclerotic plaque formation and progression. Along these lines, the accelerated atherosclerosis in diabetic ApoE^−/−^ mice was preceded by elevated blood monocytes and higher expression of endothelial activation- and inflammatory markers in the aorta. Already after 4 weeks of diabetes, a time-point when no changes in aortic plaque size had yet occurred, expression of VCAM-1, MCP-1, IL-1β, Cox2, TF and maybe also IL-6 and ICAM-1 (borderline significance) were higher than in control non-diabetic mice. The enhanced pro-inflammatory burden in diabetic mice is also reflected by the overall increased levels of circulating plasma cytokines (IL-6, IFN-γ, IL-12p70, IL-1β, IL-10, TNFα, OPN and sVCAM-1; Figure 5) after 8 weeks of diabetes. One important observation in this study was that A-285222 treatment for 4 weeks significantly reduced the diabetes-driven IL-6 levels in plasma as well as mRNA expression in the aortic arch. IL-6 is one of the most prominent pro-inflammatory cytokines, extensively studied in the context of atherogenesis [37]. It can be generated locally by cells within the lesions or released by adipose tissue into the circulation, promoting endothelial dysfunction, VSMC proliferation and migration as well as recruitment and activation of inflammatory cells, hence amplifying the inflammatory response. Moreover, IL-6 stimulates the expression of scavenger receptors SR-A and CD36, involved in the uptake of modified LDL and formation of foam cells [38]. Lack of this positive stimulation due to reduced IL-6 levels could explain the reduced plaque lipids observed in diabetic mice after A-285222 treatment (Figures 2 and 3A). The reduced IL-6 expression after treatment with A-285222 is in line with previous studies by us and other investigators, showing NFAT-dependent regulation of IL-6 gene expression in VSMCs [10], [39] and in human resistance arteries [7].

The NFAT blocker A-285222 belongs to a series of 3,5-bis (trifluoromethyl)pyrazole (BTP) derivatives originally developed in a search for safer immunosuppressive drugs. These drugs maintain NFAT in a phosphorylated state, blocking its nuclear import and subsequent transcription, without affecting NF-κB or AP-1 activation, or calcineurin phosphatase activity [40]. *In vivo* administration of A-285222 completely blocked diabetes-induced NFAT-transcriptional activity in the aorta, leading to reduced expression of IL-6, OPN, MCP-1, ICAM-1, CD68 and TF, all established players in atherogenesis, as well as to reduced diabetes-induced atherosclerosis. This was achieved without any effect on body weight, blood glucose or lipid levels and at concentrations that had no impact on NFAT activity in spleen or thymus, on T-cell proliferation rates or cytokine secretion capacity, ruling out systemic immunosuppression as the mechanism behind reduced atherosclerosis. A-285222 did not affect the number or phenotype of circulating blood monocytes, nor did it alter the numbers of T-regulatory cells in the spleen. The reduction of TF was particularly interesting, given the lack of available systemic strategies that target TF expression [41]. The dose of A-285222 used here and plasma levels achieved upon treatment were far below those required in cynomolgus monkeys for inhibition of T-cell cytokine production, which is consistent with the negative T-cell cytokine data presented here. Furthermore, non-diabetic ApoE^−/−^ mice exhibited measurable levels of plasma cytokines, reflecting a low-grade inflammation typical of this hyperlipidemic model, however, A-285222 had no effect on these levels (Figure 5), speaking against a general immunosuppressant effect of A-285222. Together, results suggest that NFAT inhibition affects the plaque phenotype at the level of the plaque itself and not via systemic immunosuppression.

Calcineurin inhibitors (i.e. CsA and FK506) are commonly used to prevent host-versus graft disease, a therapy often associated with side effects, including increased risk of atherosclerosis. While the immunosuppressive effects of these drugs are directly related to the inhibition of NFAT in immune cells, the adverse cardiovascular effects seem to be NFAT-independent and mediated via intracellular cyclophilin and chaperone activities, extracellular cyclophilin A and NFAT-independent transcriptional effects [42]. The degrees to which these NFAT-independent pathways are engaged seem to be dose-dependent [43]. Low-dose FK506 (∼0.2 ng/mL) inhibited collar-induced atherosclerosis progression and promoted plaque stability in ApoE^−/−^ mice, whereas higher doses similar to those given to transplant patients engaged instead NF-κB in macrophages and consequently increased production of cytokines. Other serious side effects associated with CsA treatment are hyperlipidemia and diabetes [42]; but these were not observed after treatment with A-285222.

Despite major advances in the treatment of cardiovascular disease during the past decades, with the introduction of lipid lowering, anti-thrombotic and anti-hypertensive drugs, there is still no available therapy that specifically targets macrovascular diabetic complications. Our data reveals the NFAT-signaling pathway as a promising target for the treatment of accelerated atherosclerosis in diabetes.

## Supporting Information

Figure S1 **Diabetes increases atherosclerosis in the descending aorta, but the overall plaque area is lower than in the aortic arch.** Summarized data from measurements of en face lesion area in the descending aorta for comparison with the aortic arch data from the same animals included in Figure 2. Results are from control and diabetic female ApoE−/− mice that had been treated for 4 weeks with the NFAT blocker A-285222 or saline. Mice were 30 weeks old at the time of analysis, performed 8 weeks after the first STZ or vehicle injection. Data is expressed as percentage of total aortic area (n = 9–12 mice/group). Two-way analysis of variance for the effect of diabetes and the drug revealed significant effect of diabetes (P<0.001). Bonferroni post-test yielded **P<0.01 vs non-diabetic saline-treated group. The inset shows corresponding data for the total aorta (i.e. arch and descending).
(PDF)

Figure S2 **Histological examination of subvalvular plaques.** (**A**) Representative cross-sections of the aortic root from control and diabetic female ApoE−/− mice treated with or without A-285222 for 4 weeks (Protocol I) stained for monocytes/macrophages (Moma-2), collagen, α-smooth muscle actin (α-SMA), tissue factor (TF, red) and osteopontin (OPN, red). Moma-2 and α-SMA stained sections were counter-stained with Harris hematoxylin; TF and OPN stained sections were counter-stained with SYTOX Green. Scale = 500 µm (Moma-2); = 100 µm (collagen, TF, OPN); = 50 µm (α-SMA). (**B, C**) Summarized data from confocal immunofluorescence experiments showing mean fluorescence intensity for plaque TF and OPN. Three to six sections for each animal were analyzed (n = 9–12 mice/group). Two-way ANOVA revealed significant effect of diabetes on OPN expression (P<0.0001). Bonferroni post-test yielded *P<0.05 and **P<0.01 vs corresponding non-diabetic groups.
(PDF)

Figure S3 **Lipid deposition in the liver is not affected by NFAT inhibition.** (**A**) Representative liver sections from diabetic female ApoE−/− mice treated with or without A-285222 for 4 weeks (Protocol I) were stained with hematoxylin-eosin (H&E) and oil red O (ORO). Scale = 100 µm. (**B**) Lipid deposition in the liver was evaluated from three ORO-stained sections per mouse using computer-assisted image analysis (n = 6–7 mice/group).
(PDF)

Figure S4 **High fat diet does not affect NFAT-dependent transcriptional activity in the aorta.** NFAT-luciferase activity in the thoracic aorta from mice fed normal chow diet (white bars) or a high fat diet (grey bars) during 4 or 8 weeks (Protocol III). Values are expressed as RLU per µg protein (n = 7–12 mice/group).
(PDF)

Figure S5 **Diabetes leads to increased expression of inflammatory and endothelial activation markers in the aortic arch.** Gene expression analyses by qRT-PCR in the aortic arch of control and diabetic ApoE−/− mice analyzed after 4 weeks of diabetes (Protocol I, n = 7–10 mice/group). HPRT and β-actin were used as endogenous controls. Data (Rel. mRNA) is expressed in relation to diabetic mice. *P<0.05 and **P<0.01.
(PDF)

Figure S6 **In vivo A-285222 treatment does not affect splenocyte proliferative capacity.** (**A–B**) [Methyl-3H]thymidine incorporation (counts per minute, cpm) after stimulation with or without anti-CD3/CD28 beads (**A**) or 2.5 µg/mL ConA (**B**) in splenocytes isolated from control and diabetic ApoE−/− mice treated for 1 week with the NFAT blocker A-285222 or saline (Protocol I; n = 9–11 mice/group) (**C**). Proliferation after stimulation with or without 2.5 µg/mL ConA in splenocytes from control mice treated for 4 weeks with the NFAT blocker A-285222 or saline (Protocol IV; n = 12–13 mice/group). (**D**) Flow cytometry data showing percentages of CD4+CD25+Foxp3+ regulatory T-cells (of total CD3+ splenocytes) in the same mice as in A. Two-way ANOVA revealed significant effect of diabetes (P<0.001). Bonferroni post-test yielded *P<0.05 vs. corresponding non-diabetic groups. (**E**) Percentages of CD3+CD4+D8- splenocytes expressing IFN-γ after stimulation with phorbol myristate acetate (PMA) and ionomycin in the same mice as in A. (**F**) Representative spleen sections stained for hematoxylin-eosin and **(G)** spleen weight in relation to tibia length from the same mice as in A. Scale = 500 µm.
(PDF)

Figure S7 **Identification and quantification of A-285222 with GC/MS. A-285222 and the inactive analog A-216491 were identified from their mass spectra and retention indexes.** (**A**) Total ion chromatogram showing substances present in a plasma sample from a mouse injected i.c. with 1.5 mg A-285222 per kg body weight. (**B**) Reconstructed ion chromatogram from the same sample as in A, showing retention indexes (s) of A-216491 (left peak, m/z = 295) and A-285222 (right peak, m/z = 416). (**C**) Mass spectra for A-285222 including the molecular ion (m/z = 416). Only the molecular ion was selective and used for quantification. (**D**) Quantification of A-285222 in plasma samples collected at different time points after i.p. injection of 0.29 mg A-285222 per kg body weight (n = 2–4 mice/time point).
(PDF)

Table S1 **Effects of diabetes and A-285222 on splenocyte cytokine production.** Splenocytes were isolated from control and diabetic mice that had been treated for 1 week with the NFAT blocker A-285222 or saline (Protocol I; n = 8–11 mice/group) after which they were cultured either under control non-stimulated conditions or with anti-CD3/CD28 beads for 48 hours. Levels of interferon (IFN)-γ, interleukin (IL)-1β, IL-2, IL-4, IL-5, KC/GRO (keratinocyte chemoattractant; keratinocyte-derived chemokine/growth related oncogene), IL-10, IL-12total and tumor necrosis factor (TNF)-α. were measured in the culture media collected at the end of the experiments. Data was analyzed by two-way ANOVA (for the effects of diabetes and A-285222). Values represent mean ± SD; significant differences after Bonferroni post-tests are indicated in the table. Diabetes had no impact on the levels of cytokines produced by non-stimulated splenocytes, while A-285222 treatment resulted in decreased IFN-γ and IL-2 (both P<0.05). Bonferroni post-test yielded *P<0.05 only for IL-2. CD3/CD28 stimulated cells from diabetic mice produced lower levels of IFN-γ (P<0.05) and IL-5 (P<0.01) but higher TNF-α. (P<0.05). Bonferroni post-test yielded #P<0.05 for TNF-α. A-285222 treatment had no impact on the ability of splenocytes to respond to CD3/CD28 stimulation.
(PDF)
