# Supplementary figures and images for: Inhibition of Nuclear Factor of Activated T-Cells (NFAT) Suppresses Accelerated Atherosclerosis in Diabetic Mice
Source: PLoS One. 2013 Jun 3;8(6):e65020. doi: 10.1371/journal.pone.0065020 (PMC3670844; doi:10.1371/journal.pone.0065020)

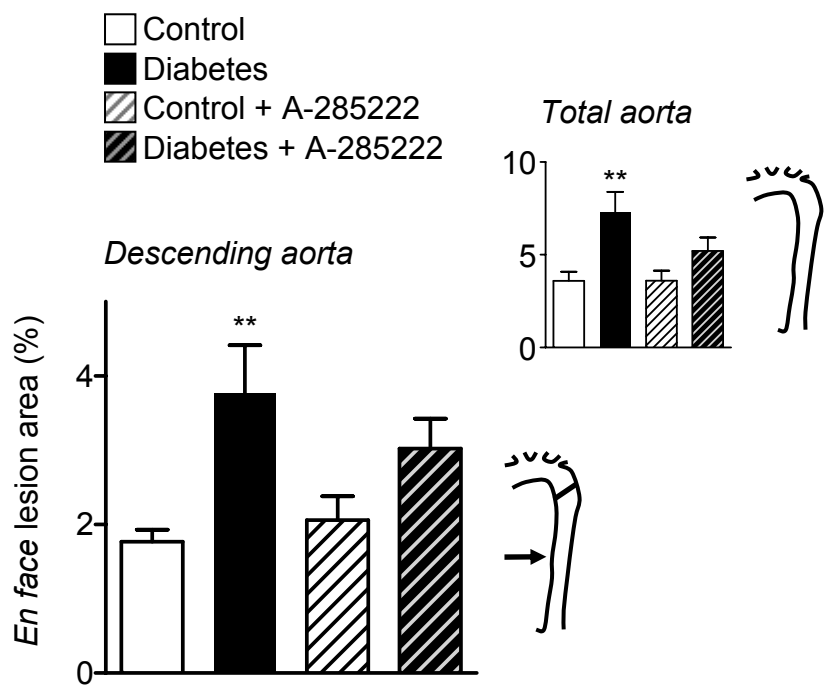

**Figure S1**

Supplement: Figure S1 — Diabetes increases atherosclerosis in the descending aorta, but the overall plaque area is lower than in the aortic arch. Summarized data from measurements of en face lesion area in the descending aorta for comparison with the aortic arch data from the same animals included in Figure 2. Results are from control and diabetic female ApoE−/− mice that had been treated for 4 weeks with the NFAT blocker A-285222 or saline. Mice were 30 weeks old at the time of analysis, performed 8 weeks after the first STZ or vehicle injection. Data is expressed as percentage of total aortic area (n = 9–12 mice/group). Two-way analysis of variance for the effect of diabetes and the drug revealed significant effect of diabetes (P<0.001). Bonferroni post-test yielded **P<0.01 vs non-diabetic saline-treated group. The inset shows corresponding data for the total aorta (i.e. arch and descending). (PDF) [file pone.0065020.s001.pdf]

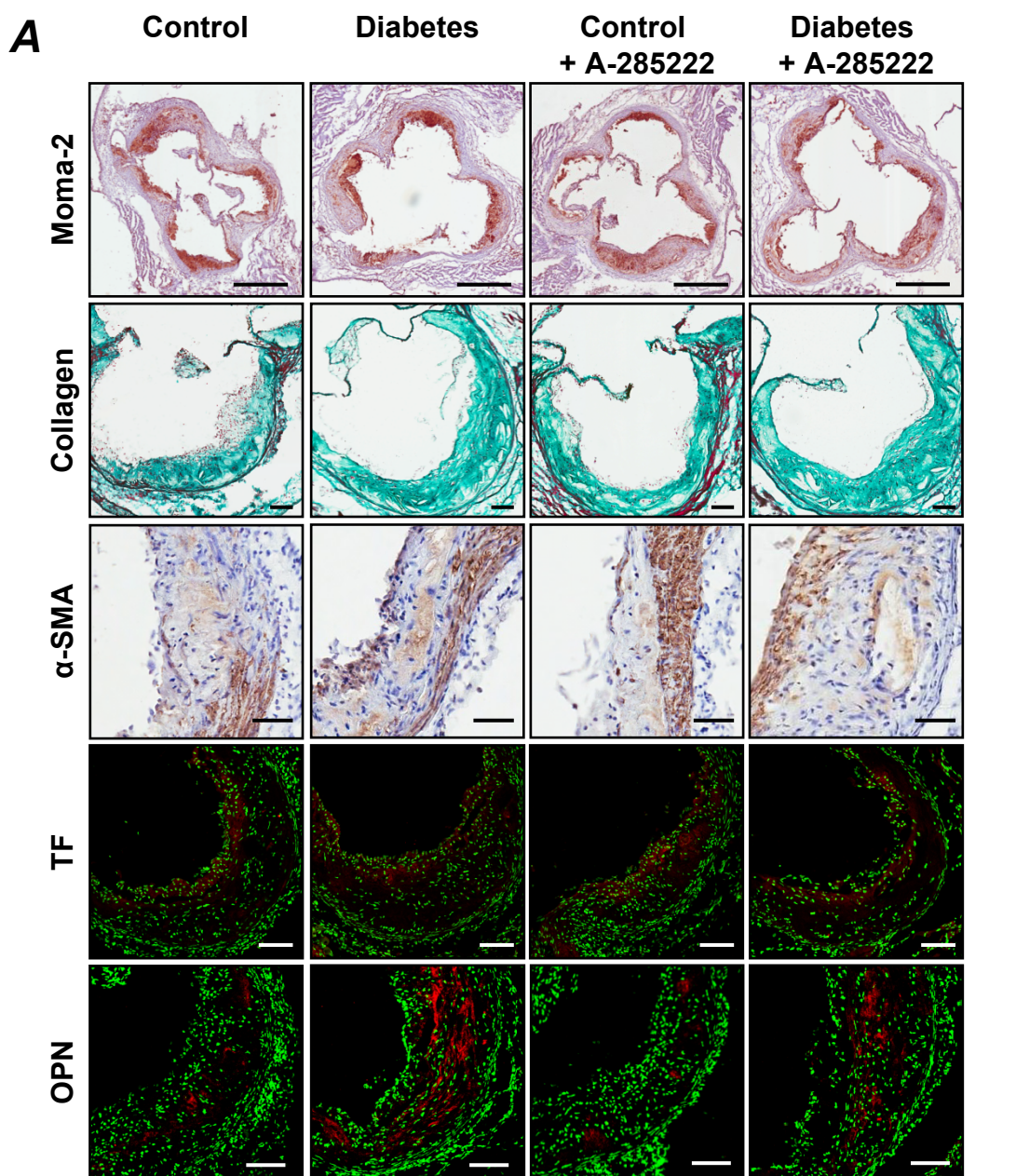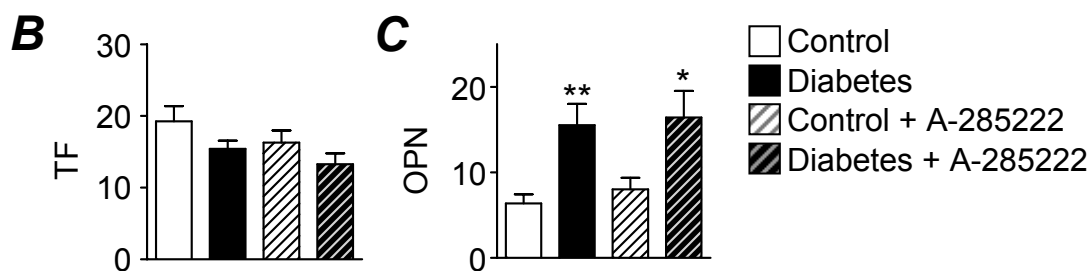

**Figure S2**

Supplement: Figure S2 — Histological examination of subvalvular plaques. (A) Representative cross-sections of the aortic root from control and diabetic female ApoE−/− mice treated with or without A-285222 for 4 weeks (Protocol I) stained for monocytes/macrophages (Moma-2), collagen, α-smooth muscle actin (α-SMA), tissue factor (TF, red) and osteopontin (OPN, red). Moma-2 and α-SMA stained sections were counter-stained with Harris hematoxylin; TF and OPN stained sections were counter-stained with SYTOX Green. Scale = 500 µm (Moma-2); = 100 µm (collagen, TF, OPN); = 50 µm (α-SMA). (B, C) Summarized data from confocal immunofluorescence experiments showing mean fluorescence intensity for plaque TF and OPN. Three to six sections for each animal were analyzed (n = 9–12 mice/group). Two-way ANOVA revealed significant effect of diabetes on OPN expression (P<0.0001). Bonferroni post-test yielded *P<0.05 and **P<0.01 vs corresponding non-diabetic groups. (PDF) [file pone.0065020.s002.pdf]

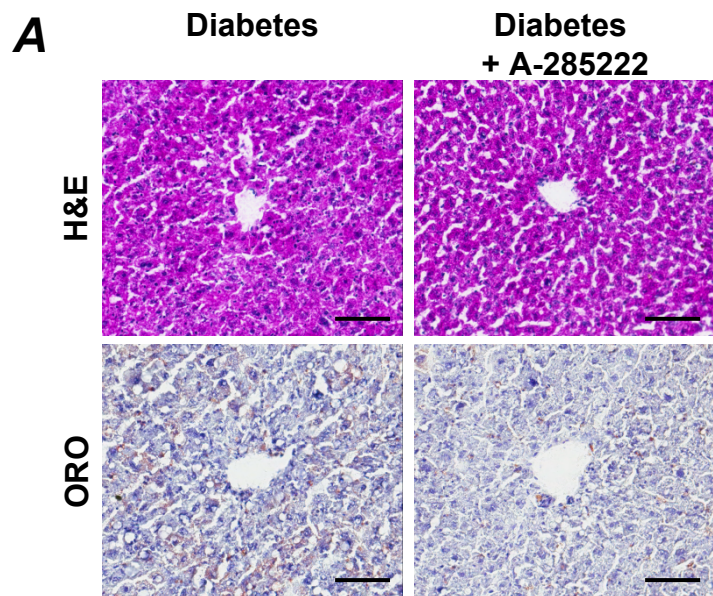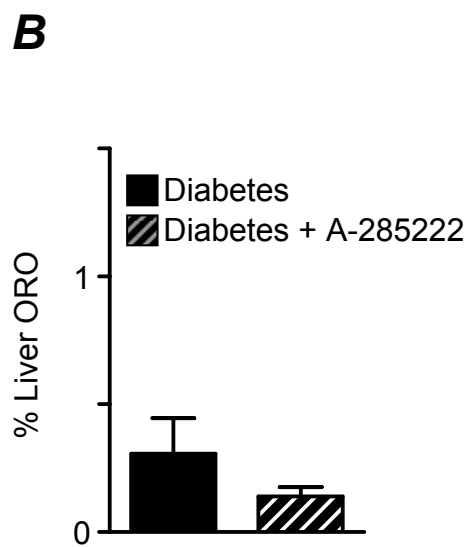

**Figure S3**

Supplement: Figure S3 — Lipid deposition in the liver is not affected by NFAT inhibition. (A) Representative liver sections from diabetic female ApoE−/− mice treated with or without A-285222 for 4 weeks (Protocol I) were stained with hematoxylin-eosin (H&E) and oil red O (ORO). Scale = 100 µm. (B) Lipid deposition in the liver was evaluated from three ORO-stained sections per mouse using computer-assisted image analysis (n = 6–7 mice/group). (PDF) [file pone.0065020.s003.pdf]

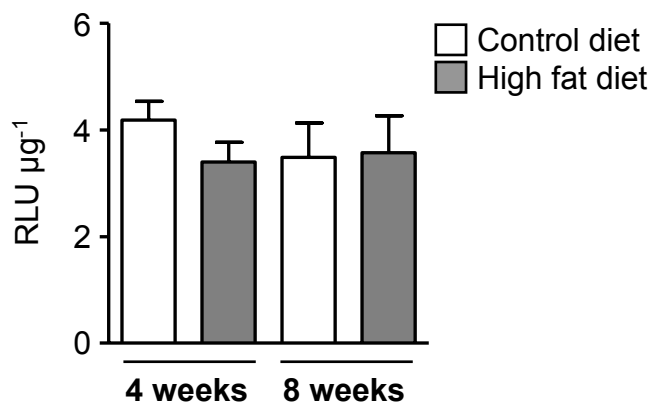

**Figure S4**

Supplement: Figure S4 — High fat diet does not affect NFAT-dependent transcriptional activity in the aorta. NFAT-luciferase activity in the thoracic aorta from mice fed normal chow diet (white bars) or a high fat diet (grey bars) during 4 or 8 weeks (Protocol III). Values are expressed as RLU per µg protein (n = 7–12 mice/group). (PDF) [file pone.0065020.s004.pdf]

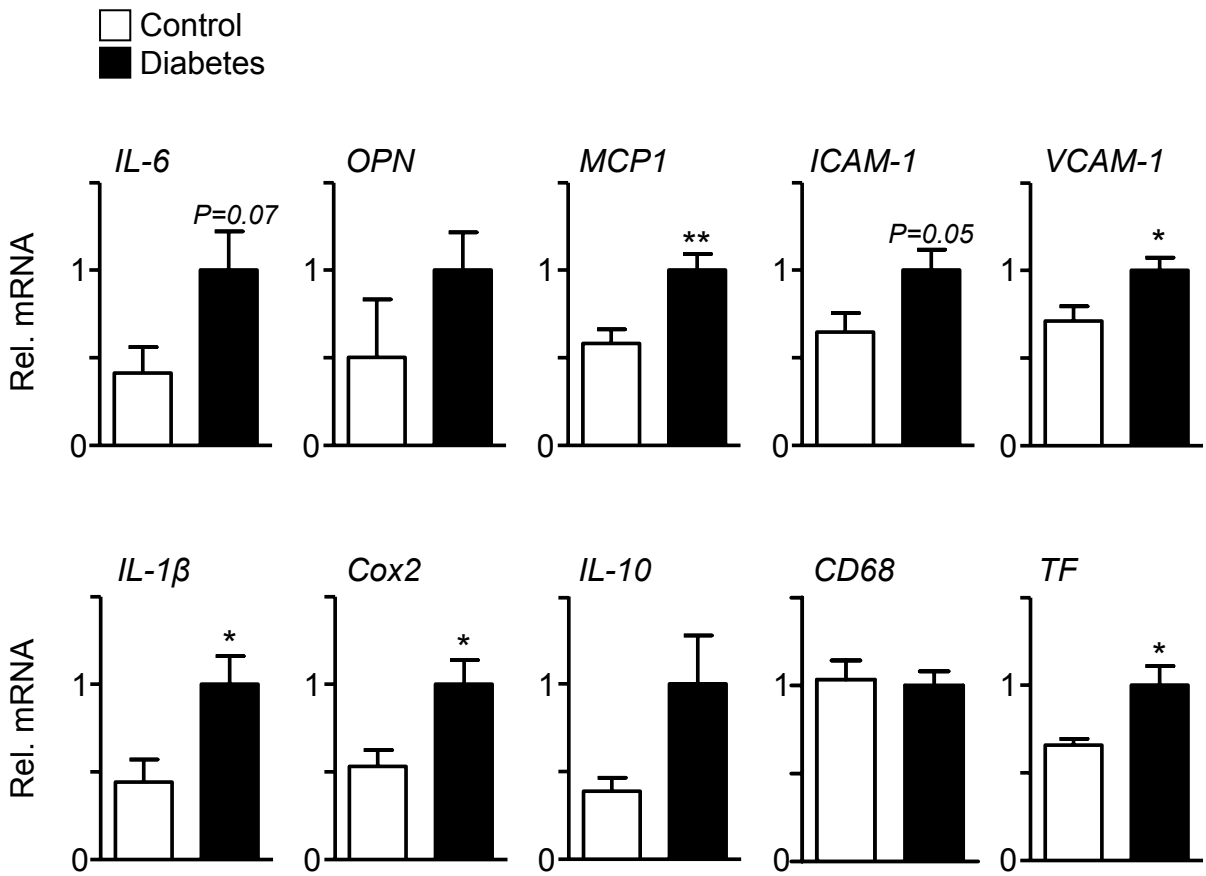

**Figure S5**

Supplement: Figure S5 — Diabetes leads to increased expression of inflammatory and endothelial activation markers in the aortic arch. Gene expression analyses by qRT-PCR in the aortic arch of control and diabetic ApoE−/− mice analyzed after 4 weeks of diabetes (Protocol I, n = 7–10 mice/group). HPRT and β-actin were used as endogenous controls. Data (Rel. mRNA) is expressed in relation to diabetic mice. *P<0.05 and **P<0.01. (PDF) [file pone.0065020.s005.pdf]

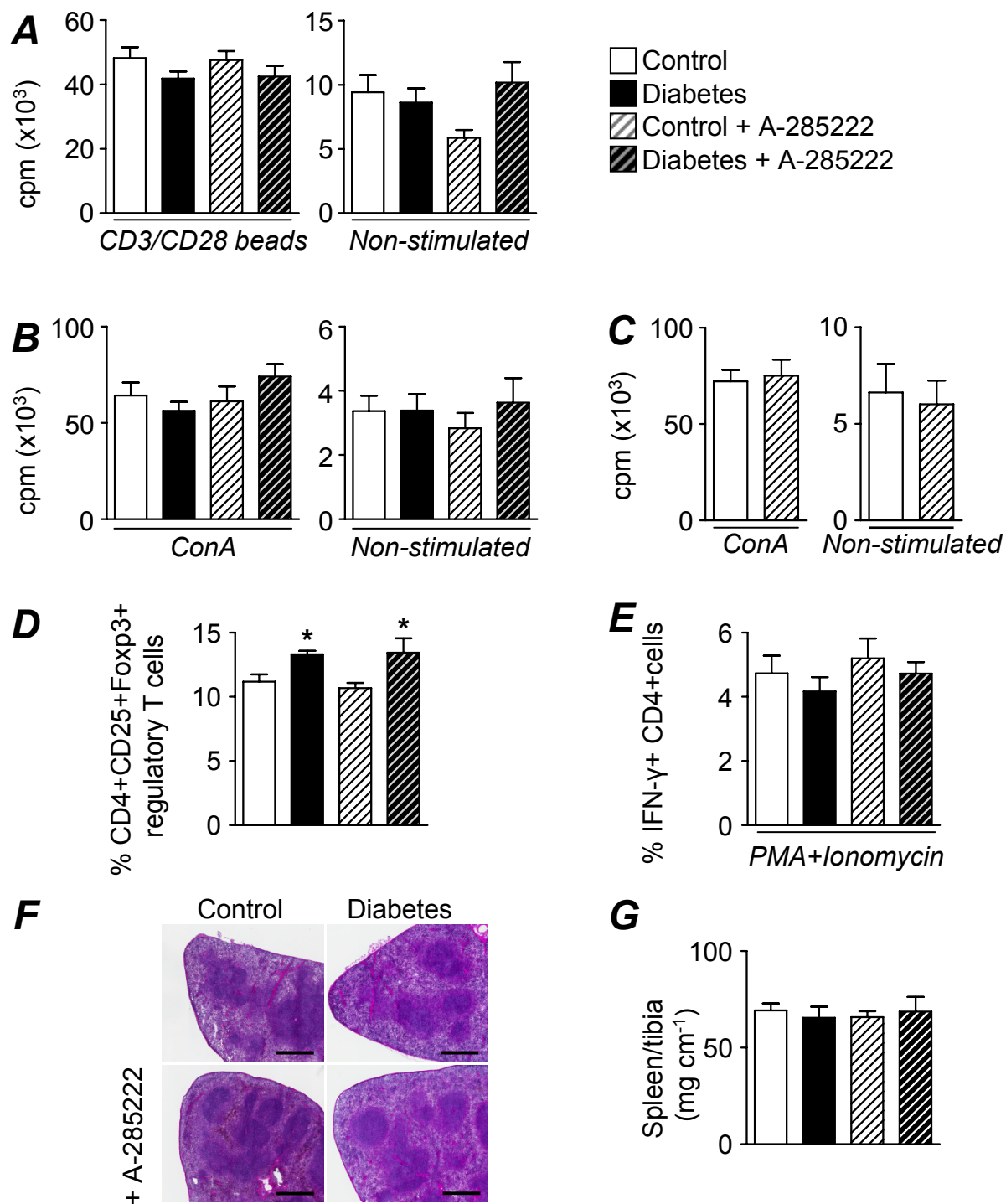

**Figure S6**

Supplement: Figure S6 — In vivo A-285222 treatment does not affect splenocyte proliferative capacity. (A–B) [Methyl-3H]thymidine incorporation (counts per minute, cpm) after stimulation with or without anti-CD3/CD28 beads (A) or 2.5 µg/mL ConA (B) in splenocytes isolated from control and diabetic ApoE−/− mice treated for 1 week with the NFAT blocker A-285222 or saline (Protocol I; n = 9–11 mice/group) (C). Proliferation after stimulation with or without 2.5 µg/mL ConA in splenocytes from control mice treated for 4 weeks with the NFAT blocker A-285222 or saline (Protocol IV; n = 12–13 mice/group). (D) Flow cytometry data showing percentages of CD4+CD25+Foxp3+ regulatory T-cells (of total CD3+ splenocytes) in the same mice as in A. Two-way ANOVA revealed significant effect of diabetes (P<0.001). Bonferroni post-test yielded *P<0.05 vs. corresponding non-diabetic groups. (E) Percentages of CD3+CD4+D8- splenocytes expressing IFN-γ after stimulation with phorbol myristate acetate (PMA) and ionomycin in the same mice as in A. (F) Representative spleen sections stained for hematoxylin-eosin and (G) spleen weight in relation to tibia length from the same mice as in A. Scale = 500 µm. (PDF) [file pone.0065020.s006.pdf]

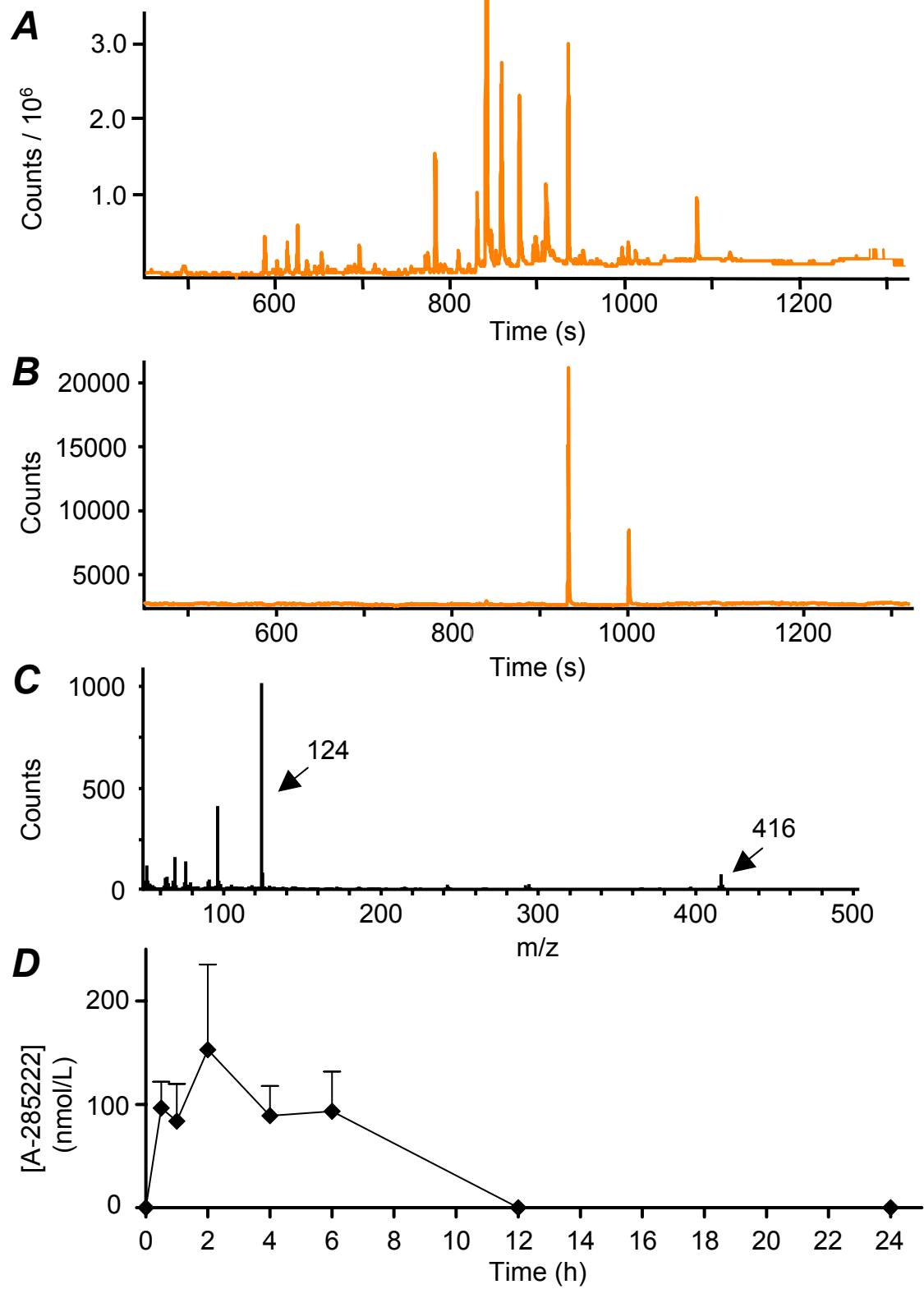

**Figure S7**

Supplement: Figure S7 — Identification and quantification of A-285222 with GC/MS. A-285222 and the inactive analog A-216491 were identified from their mass spectra and retention indexes. (A) Total ion chromatogram showing substances present in a plasma sample from a mouse injected i.c. with 1.5 mg A-285222 per kg body weight. (B) Reconstructed ion chromatogram from the same sample as in A, showing retention indexes (s) of A-216491 (left peak, m/z = 295) and A-285222 (right peak, m/z = 416). (C) Mass spectra for A-285222 including the molecular ion (m/z = 416). Only the molecular ion was selective and used for quantification. (D) Quantification of A-285222 in plasma samples collected at different time points after i.p. injection of 0.29 mg A-285222 per kg body weight (n = 2–4 mice/time point). (PDF) [file pone.0065020.s007.pdf]
