# Supplementary material for: Inhibition of Nuclear Factor of Activated T-Cells (NFAT) Suppresses Accelerated Atherosclerosis in Diabetic Mice
Source: PLoS One. 2013 Jun 3;8(6):e65020. doi: 10.1371/journal.pone.0065020 (PMC3670844; doi:10.1371/journal.pone.0065020)
Supplement: Table S1 — Effects of diabetes and A-285222 on splenocyte cytokine production. Splenocytes were isolated from control and diabetic mice that had been treated for 1 week with the NFAT blocker A-285222 or saline (Protocol I; n = 8–11 mice/group) after which they were cultured either under control non-stimulated conditions or with anti-CD3/CD28 beads for 48 hours. Levels of interferon (IFN)-γ, interleukin (IL)-1β, IL-2, IL-4, IL-5, KC/GRO (keratinocyte chemoattractant; keratinocyte-derived chemokine/growth related oncogene), IL-10, IL-12total and tumor necrosis factor (TNF)-α. were measured in the culture media collected at the end of the experiments. Data was analyzed by two-way ANOVA (for the effects of diabetes and A-285222). Values represent mean ± SD; significant differences after Bonferroni post-tests are indicated in the table. Diabetes had no impact on the levels of cytokines produced by non-stimulated splenocytes, while A-285222 treatment resulted in decreased IFN-γ and IL-2 (both P<0.05). Bonferroni post-test yielded *P<0.05 only for IL-2. CD3/CD28 stimulated cells from diabetic mice produced lower levels of IFN-γ (P<0.05) and IL-5 (P<0.01) but higher TNF-α. (P<0.05). Bonferroni post-test yielded #P<0.05 for TNF-α. A-285222 treatment had no impact on the ability of splenocytes to respond to CD3/CD28 stimulation. (PDF) [file pone.0065020.s008.pdf]

TABLE S1.

Cytokine (pg/mL)	Control (n=11)	Diabetes (n=11)	Control + A-285222 (n=10)	Diabetes + A-285222 (n=8)
Non-stimulated				
IFN-γ	1.36±1.22	1.06±0.30	0.78±0.56	0.33±0.17
IL-1β	0.62±0.54	0.45±0.38	0.41±0.28	0.80±0.35
IL-2	33.0±23.8	18.7±11.1	15.3±8.9*	12.1±7.04
IL-4	1.04±0.87	0.87±0.83	0.40±0.55	0.70±0.61
IL-5	0.20±0.17	0.24±0.31	0.25±0.25	0.21±0.12
KC/GRO	3.25±1.90	3.24±2.14	4.52±4.12	4.70±2.69
IL-10	11.9±8.63	12.2±5.73	11.1±10.5	13.3±4.24
IL-12total	184±84.8	190±116	148±74.5	160±36.5
TNF-α	0.43±0.86	0.79±1.51	0.26±0.62	0.29±0.54
CD3/CD28 stimulated				
IFN-γ	4197±941	3523±1110	4202±823	3408±889
IL-1β	23.4±12.0	25.2±16.9	28.2±11.6	24.0±21.4
IL-2	74.2±28.1	106.2±44.3	116.9±47.4	92.8±35.4
IL-4	31.4±11.2	35.9±22.6	46.4±15.2	30.8±20.4
IL-5	62.1±39.3	35.9±14.4	58.6±35.9	29.7±11.6
KC/GRO	32.0±9.37	39.1±19.8	38.4±10.4	44.4±27.0
IL-10	282±129	203±59.3	242±65.0	214±78.1
IL-12total	287±89.3	269±93.1	243±56.6	256±67.0
TNF-α	110.7±31.0	125.2±33.5	98.4±37.7	138.5±42.0#
